# Cognition and motor phenotypes in ALS: a retrospective study

**DOI:** 10.1007/s10072-022-06157-x

**Published:** 2022-05-24

**Authors:** Edoardo Nicolò Aiello, Debora Pain, Alice Radici, Kalliopi Marinou Aktipi, Riccardo Sideri, Ildebrando Appollonio, Gabriele Mora

**Affiliations:** 1 Ph.D. Program in Neuroscience, School of Medicine and Surgery, University of Milano-Bicocca, Via Cadore 48, 20900 Monza, Italy; 2 Istituti Clinici Scientifici Maugeri, IRCCS Milano, Milan, Italy; 3 Neurology Section, School of Medicine and Surgery, University of Milano-Bicocca, Monza, Italy

**Keywords:** Amyotrophic lateral sclerosis, Frontotemporal degeneration, Cognitive impairment: Upper motor neuron, Lower motor neuron

## Abstract

**Background:**

Amyotrophic lateral sclerosis (ALS) is phenotypically heterogeneous in motor manifestations, and the extent of upper vs. lower motor neuron involvement is a widespread descriptor. This study aimed to examine cognition across different ALS motor phenotypes.

**Methods:**

ALS patients (*N* = 124) were classified as classical (*N* = 66), bulbar (*N* = 13), predominant-upper motor neuron (PUMN; *N* = 19), and predominant-lower motor neuron (PLMN; *N* = 26) phenotypes. Cognition was assessed with the Edinburgh Cognitive and Behavioural ALS Screen (ECAS) and function with the ALS Functional Rating Scale—Revised (ALSFRS-R). Revised ALS-FTD consensus criteria were applied for cognitive/behavioral phenotyping.

**Results:**

Defective ECAS-total scores were detected in all groups — bulbar: 15.4%, classical: 30.3%, PLMN: 23.1%, and PUMN: 36.8%. Classical and PUMN ALS patients performed worse than PLMN ones on ECAS-total, ALS-specific, Fluency, and Executive measures. No other difference was detected. Worse ASLFRS-R scores correlated with poorer ECAS-total scores in classical ALS patients.

**Conclusions:**

Frontotemporal cognitive deficits are more prevalent in PUMN and classical ALS and linked to disease severity in the latter, but occur also in PLMN phenotypes.

**Supplementary information:**

The online version contains supplementary material available at 10.1007/s10072-022-06157-x

## Introduction

Amyotrophic lateral sclerosis (ALS) is phenotypically heterogeneous in motor manifestations, with the extent of upper vs. lower motor neuron involvement being a widespread descriptor [1]. Besides classical ALS, which affects the whole disynaptic motor pathway, predominant-upper or -lower motor neuron phenotypes are indeed acknowledged (PUMN; PLMN), although less prevalent/incident [2].

In respect to cognitive features, involvement within the spectrum of frontotemporal degenerations (FTDs) — i.e., dysexecutive features and language deficits — has been ascertained to occur in up to 50% of classical ALS patients [3].

However, less attention has been given to cognition in patients with PUMN/PLMN ALS phenotypes [2], although emerging genetic [4], histological [5], radiological [6], and clinical [7–10] evidence suggest that they may likewise present with frontotemporal pathology. In such a framework, a common pathophysiological mechanism would account for cognitive involvement across different ALS motor phenotypes [11]. Moreover, as to the association between cognitive and motor features in ALS, it has been postulated that patients with bulbar phenotypes [2] are at higher risk for frontotemporal involvement [12].

Nevertheless, few studies have to date focused on exploring cognition across different motor phenotypes of ALS [13], notwithstanding the prognostic relevance of cognitive assessment in this population [14]. Such investigations are indeed crucial to determine whether ALS patients with atypical, PUMN/PLMN phenotypes likewise show frontotemporal features, and, in turn, to raise the awareness as to the need for screening for cognition in these patients.

Given the above premises, this study thus aimed to examine cognition in PUMN and PLMN ALS as compared to both classical and bulbar phenotypes.

## Methods

### Participants

Data from *N* = 124 ALS patients referred to Istituti Clinici Scientifici Maugeri, IRCCS Milano, Italy between 2016 and 2021 were retrospectively retrieved.

Patients had no history of (1) other neurological/neuropsychiatric disorders; (2) uncorrected visual/hearing deficits; and (3) severe, uncompensated metabolic/internal conditions and organ/systemic failures.

Based on clinical and instrumental examinations, motor phenotypes were defined, by two neurologists with long-lasting expertise in ALS motor phenotyping (K. M. A. and G. M.), as (1) classical ALS (*N* = 66); (2) bulbar ALS (*N* = 13); (3) PUMN ALS (*N* = 19); and (4) PLMN ALS (*N* = 26) [2]. Bulbar ALS patients (*N* = 13) were addressed as a separate group since the degree of UMN vs. LMN involvement could not be estimated based on the adopted phenotyping system [2].

According to Strong’s revised criteria [3], patients were classified as either cognitively (ALSci), behaviorally (ALSbi), cognitively and behaviorally (ALScbi) impaired, or ALS-FTD based on a thorough neuropsychological evaluation encompassing measures of both instrumental (i.e., language, memory, praxis, visuo-spatial abilities) and non-instrumental (i.e., executive functioning and attention) cognitive functions, as well as of FTD-like behavioral features (Supplementary Table 1). Strong classifications were performed within the routine clinical practice by neuropsychologists with long-lasting expertise in cognitive assessment of ALS patients (D. P. and A. R.).

This study was approved by the Ethics Committee of Istituti Clinici Scientifici Maugeri (I.D.: 2495 CE, 12/01/2021).

### Materials

Cognition was assessed by means of the Edinburgh Cognitive and Behavioural ALS Screen (ECAS) [15], while functional outcome via the ALS Functional Rating Scale - Revised (ALSFRS-R) [16]. The ECAS (range: 0–136) assesses both an ALS-specific (Executive, Fluency, and Language sub-scales; range: 0–100) and ALS-nonspecific functions (Memory and Visuo-spatial sub-scales; range: 0–36), by controlling for motor disabilities (dysarthria/upper limb impairment).

### Statistics

ECAS scores were heavily left-skewed (ceiling effect) and overdispersed (high inter-individual variability), as evidenced by high skewness and kurtosis values (≥|1| and |3|, respectively), visual abnormalities at histograms and quantile-quantile plots, and significant statistics at Shapiro Wilk’s test [17]. Therefore, instead of linear models, Negative Binomial regressions were performed to test the effect of motor phenotypes on the ECAS-total and its sub-scores [18]. In order for them to be modeled by the Negative Binomial, which accounts for right-skewed, overdispersed count-like data, the number of errors, instead of the score out of a maximum, was addressed as the outcome operationalizing accuracy [18]. Such a statistical approach has proved to be effective in modeling cognitive data of patients with neurological conditions [19, 20], including ALS [20].

According to sample size estimation procedures for Negative Binomial models suggested by Cundill & Alexander [21], the minimum sample size for each group (i.e., bulbar, classical, PLMN, and PUMN) was set at *N* = 11 (total *N* of 44), by addressing an expected variability in ECAS-total scores of 15% across phenotypes, an overdispersion parameter *k* of 2 for each phenotype, and a power 1 − β of 95%. Within this computation, type-I error level (*α* = .05) was Bonferroni-adjusted (4 groups, 6 comparisons; *α*_adjusted_ = .008) since Cundill & Alexander’s procedure [21] was designed for comparing two means only.

Age, education, sex, disease duration, disease severity (ALSFRS-R), presence of bulbar signs, presence of C9orf72 repeat expansion, and Strong’s diagnoses were covaried within each model. Covarying for Strong’s diagnoses was deemed as fundamental in order to remove error variance possibly entered into ECAS scores by the different severity and nature of neuropsychological dysfunctions of patients, which cannot be fully accounted for by the ECAS, as it being a screening measure. The significance level (*α* = .05) was corrected via Bonferroni’s method for multiple comparisons. Analyses were performed with jamovi 1.6 (https://www.jamovi.org/) and SPSS 27 (IBM Corp., 2021).

## Results

Table 1 shows patients’ background, clinical, and cognitive measures. Forced vital capacity (FVC) data were missing completely-at-random for 53 patients. Defective scores on both the ECAS total and its sub-scores were detected across the four groups.

**Table 1:** Patients’ demographic, clinical, and cognitive measures

Phenotypes	Bulbar ALS	Classical ALS	PLMN ALS	PUMN ALS
*N*	13	66	26	19
Sex (M/F)	4/9	32/34	14/12	9/10
Age (years)	67.62 ± 10.57 (51–83)	62.6 ± 11.1 (30–82)	64 ± 12.2 (37–81)	66.2 ± 10.1 (50–84)
Education (years)	11 ± 4.18 (5–17)	11.2 ± 4.1 (4–19)	11 ± 4.8 (5–25)	11.6 ± 2.3 (5–18)
ALSFRS-R	28.83 ± 9.24 (9–44)	28.6 ± 9.4 (8–46)	27.8 ± 17.7 (4–46)	29.6 ± 7.8 (15–41)
Disease duration (months)	26.18 ± 19.38 (5.77–62.53)	32.1 ± 31.2 (4.5–165.9)	52.4 ± 44.9 (7.2–210.2)	84.9 ± 81.8 (3.6–274.6)
Diagnostic delay (months)	11.5 ± 9.12 (.7–29.8)	16.3 ± 18.5 (2.8–103.7)	20.9 ± 17.7 (2.1–66.4)	46.2 ± 61.6 (3.1–251.6)
Bulbar signs (%)	-	51.5%	38.5%	68.4%
FVC (%)^a^	92.28 ± 49.69 (41–175)	84.17 ± 30.94 (29–170)	90.83 ± (44.5–125)	79.73 ± 29.63 (50–140)
Genetics (*N*)				
C9orf72	-	6	-	-
SOD1	-	1	2	-
TARDP	1	-	-	1
Familiarity	-	-	-	1
Strong et al. (2017) classifications				
CN (%)	53.8%	68.2%	65.4%	57.9%
ALSbi (%)	-	12.1%	3.8%	-
ALSci (%)	38.5%	13.6%	15.4%	31.6%
ALScbi (%)	-	1.5%	15.4%	10.5%
ALS-FTD (%)	7.7%	4.5%	-	-
ECAS				
Total	99.15 ± 16.22 (70–119)	99.1 ± 27.3 (15–129)	103.1 ± 23.1 (26–127)	91.6 ± 23.8 (39–120)
ALS-specific	72 ± 13.9 (44–88)	72.1 ± 21.5 (10–95)	76.1 ± 18.7 (17–96)	67.4 ± 18.6 (29–88)
ALS-nonspecific	26.15 ± 4.14 (18–31)	26.1 ± 6.9 (5–36)	26.8 ± 5.7 (9–34)	25.8 ± 6.7 (7–34)
Language	24.38 ± 2.76 (17–28)	23.41 ± 4.78 (8–28)	23.81 ± 4.76 (12–28)	22.53 ± 4.5 (11–28)
Executive	31.54 ± 8.34 (17–40)	33.24 ± 12.07 (0–48)	34.85 ± 9.66 (5–46)	30.47 ± 10.59 (8–41)
Fluency	16.08 ± 6.44 (2–22)	15.42 ± 6.82 (0–24)	17.46 ± 6.06 (0–24)	14.42 ± 6.35 (0–22)
Memory	15.46 ± 4.22 (7–23)	15 ± 5.911 (0–24)	15.73 ± 4.65 (4–22)	15.21 ± 5.92 (0–23)
Visuo-spatial	10.69 ± 1.49 (8–12)	11.11 ± 1.65 (5–12)	11.12 ± 1.84 (5–12)	10.63 ± 1.67 (7–12)
Below-cutoff^b^ percentage				
Total	30.8%	28.8%	23.1%	47.4%
ALS-specific	15.4%	30.3%	23.1%	36.8%
ALS-non-specific	15.4%	21.2%	7.7%	15.8%
Language	7.7%	19.7%	26.9%	26.3%
Executive	38.5%	27.3%	19.2%	36.8%
Fluency	23.1%	24.2%	15.4%	26.3%
Memory	15.4%	19.7%	7.7%	15.8%
Visuo-spatial	23.1%	13.6%	15.4.%	15.8%

*M* male, *F* female, *PUMN* predominant-upper motor neuron, *PLMN* predominant-lower motor neuron, *ALSFRS-R* Amyotrophic Lateral Sclerosis Functional Rating Scale—Revised, *CN* cognitively normal, *ALSbi* ALS with behavioral impairment, *ALSIci* ALS with cognitive impairment, *ALScbi* ALS with cognitive and behavioral impairment, *ALS-FTD* ALS with frontotemporal dementia, *ECAS* Edinburgh Cognitive and Behavioural ALS Screen

^a^Data missing for 53 patients

^b^Poletti et al*.* (2016)

Net of covariates and motor phenotypes significantly affected ECAS-total (*χ*^2^(3) = 11.22; *p* = .011), ALS-specific (*χ*^2^(3) = 11.22; *p* = .011), Executive (*χ*^2^(3) = 8.35; *p* = .039), and Fluency (*χ*^2^(3) = 8.31; *p* = .04) scores. At Bonferroni-adjusted post hoc comparisons, the error count on the ECAS-total was significantly lower for PLMN (*M* = 32.6, SE = 3.6) than PUMN patients (*M* = 46.4, *SE* = 5.86; *p* = .018). Similar results yielded as to ALS-specific scores, with PLMN patients (*M* = 22.1, SE = 2.91) performing better than classical ALS (*M* = 30.9, SE = 3.09; *p* = .02) and PUMN patients (*M* = 32.5, SE = 4.86; *p* = .038). As to Fluency sub-scores, classical ALS patients showed a higher (*p* = .028) error rate (*M* = 9.83, SE = 1.38) when compared to PLMN ones (*M* = 6.12, SE = 1.16), whereas a similar trend, although failing to reach significance, was detected as to Executive sub-scores (*p* = .056). Remaining comparisons for both ECAS-total, ALS-specific, Executive, and Fluency scores among phenotypes were not significant.

No significant phenotype effects yielded as to Language, ALS-nonspecific, Memory, and Visuo-spatial scores.

**Fig. 1:**
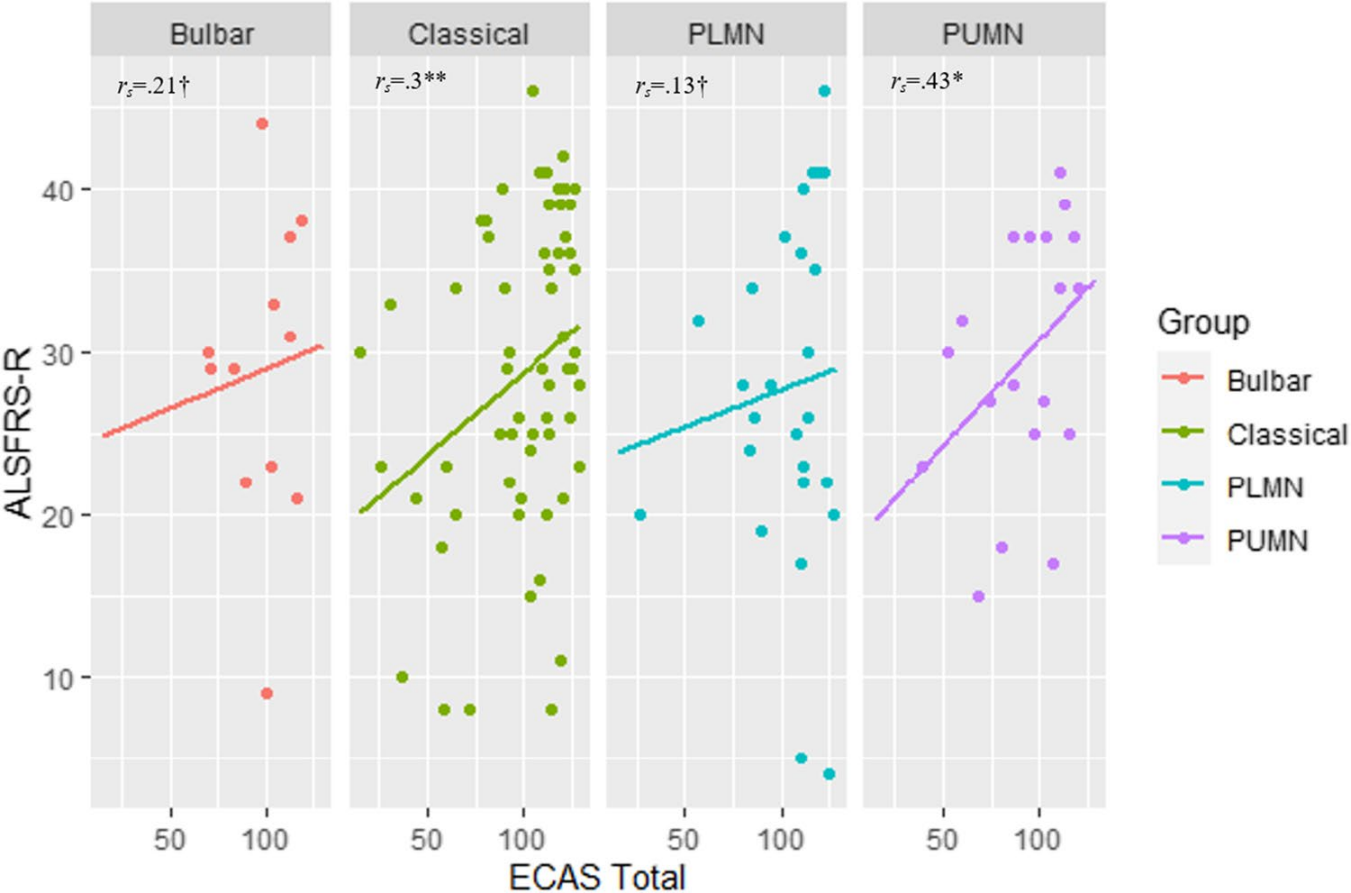
Scatterplots for the association between ALSFRS-R and ECAS-total scores paneled by ALS motor phenotypes. *ALSFRS-R* ALS Functional Rating Scale—Revised, *ECAS* Edinburgh Cognitive and Behavioural ALS Screen, *PUMN* predominant-upper motor neuron, *PLMN* predominant-lower motor neuron. **p = .019, *p = .07, ^†^*p* > .1

Interestingly, within the models on ECAS-total, ALS-specific, Language, Executive, and Fluency measures, lower ALSFRS-R scores were predictive of a higher error count (*p* ≤ .024). Therefore, to better explore this effect, Spearman’s correlations between ASLFRS-R and ECAS-total scores were run separately for each group: this association was significant in classical ALS (*r*_*s*_ (62) = .3; *p* = .019), whereas non-significant in bulbar ALS, PUMN, and PLMN patients, respectively (Fig. 1).

In order to test possible effects of FVC values, which were not included as covariates within the Negative Binomial models due to the high number of missing values, Spearman’s correlations were run with all ECAS measures. No significant association yielded at *α*_adjusted_ = .002 (*p* ≥ .121).

## Discussion

This study shows that frontotemporal involvement occurs across different motor phenotypes of ALS, although to different extents. Indeed, despite classical, bulbar, and PUMN patients being overall comparable as to their cognitive status, PLMN patients showed better cognitive outcomes when compare to classical and PUMN ALS patients. Such findings are suggestive of a more widespread cortical involvement in classical and PUMN when compared to PLMN patients [10]. However, cognitive impairment was moderately prevalent also in PLMN patients (23.1%), thus suggesting that extra-motor cortical areas may be also involved in these phenotypes [7, 8].

Moreover, at variance with previous reports [12], the present findings appear not to support the widespread notion of bulbar ALS phenotype being more strongly associated with cognitive impairment [22], since bulbar ALS patients did not differ from any of the other groups. Although caution should be exerted when interpreting the present results due to the small number of bulbar ALS patients included (*N* = 13), it is worth noting that they are in line with recent ones reporting no differences in FTD-like behavioral alterations between patients showing or not bulbar involvement [20, 23].

Notably, Strong’s criteria successfully classified not only classical and bulbar but also PUMN and PLMN patients, thus supporting their adoption to phenotypes beyond classical ALS. Consistently, ALS-specific cognitive dysfunctions, as detected by Language, Executive, and Fluency sub-scales, were present in all groups. Taken together, such findings support the notion that patients with motor phenotypes different from classical ALS likewise show cognitive changes within the spectrum of FTD [6].

In this last respect, it should be nonetheless noted that between-phenotype differences failed to emerge as to language functioning [24, 25], although it has been forwarded that this domain may be involved to a greater extent in PUMN, bulbar, and classical ALS phenotypes when compared to PLMN ones [26]. Such a finding might be due to the fact that, in accordance with evidence on the diagnostic properties of the ECAS [22], its Language sub-scale is less able to detect cognitive changes typical of ALS when compared to the Executive and Fluency sub-scales. Consistently, in the present study, the latter sub-scales were the only to yield between-phenotypes differences.

As to the associations between ALSFRS-R and ECAS scores, since the latter controls for motor disabilities [15], it is likely to reflect an actual co-variance of motor and cognitive function. This is in line with evidence on (1) a progressive cognitive decline with advancing motor impairment [27] and (2) the emerging picture of cognition being related to motor features — e.g., lateralization of motor damage [28] and hyperexcitability of motor cortices [29]. According to the present study, such associations would emerge for classical ALS only, possibly due to the fact that this phenotype entails a pervasive involvement of the motor system [2].

A number of limitations should be however listed. First, the present study did not include specific measures of upper vs. lower motor neuron involvement, at variance with the recent report by Maranzano et al. [13], who nonetheless came to similar conclusions.

Moreover, as to the association between disease severity and cognition, this work addressed only total ALSFRS-R scores: further studies are thus needed in order to unravel the interplay between specific functional domains assessed by the ALSFRS-R and the ECAS across different ALS motor phenotypes. In this respect, it has also to be acknowledged that FVC values were missing for several patients, thus prompting future works focused on a more comprehensive investigation on the association between instrumental, respiratory outcome, and cognition in ALS accounting for motor phenotypes too.

Furthermore, the present study focused only on cognitive, and not behavioral, features, which should be addressed in future investigations. Finally, it should be noted that, although certain PUMN/PLMN patients showed with lowermost ECAS scores (Table 1), no diagnoses of co-morbid FTD were posed within these two groups according to Strong’s criteria. Thereupon, albeit the ECAS has been shown to have optimal diagnostic performance against such a nosographic system [22], it is likely that, in the PUMN and PLMN groups, its consistency with Strong’s classifications might have been poorer when compared to classical and bulbar phenotypes.

In conclusion, this study shows that (1) FTD-like cognitive deficits occur across all ALS motor phenotypes; (2) cognitive impairment is more severe and/or prevalent in classical and PUMN vs. PLMN ALS phenotypes; and (3) cognitive status is linked to disease severity in classical ALS. Hence, the same attention should be given for all motor phenotypes as far as cognitive screening.

## Supplementary information

Below is the link to the electronic supplementary material.

Supplementary file1 (DOCX 19 KB)
