# Supplementary material for: Cognition and motor phenotypes in ALS: a retrospective study
Source: Neurol Sci. 2022 May 24;43(9):5397–402. doi: 10.1007/s10072-022-06157-x (PMC9385798; doi:10.1007/s10072-022-06157-x)
Supplement: Supplementary file 1 — Supplementary file1 (DOCX 19 KB) [file 10072_2022_6157_MOESM1_ESM.docx]

**Supplementary Table 1.** Neuropsychological battery for classifying ALS patients according to Strong et al. (2017) criteria.

| **Domain** | **Reference for normative data** |
| --- | --- |
| **Executive functioning** |  |
| Weigl’s Sorting Test (WST) | Spinnler, H., & Tognoni, G. (1987). *The Italian Journal of Neurological Sciences*, *6*, S1-S120. |
| Modified Wisconsin Card Sorting Test (MCST) | Caffarra, P. (2004). *Journal of Clinical and Experimental Neuropsychology*, *26*, 246-250. |
| Frontal Assessement Battery (FAB) | Appollonio, I. et al. (2005). *Neurological Sciences*, *26*, 108-116. |
| Cognitive Estimation Task (CET) | Della Sala, S. et al. (2003). *Neurological Sciences*, *24*, 10-15. |
| Coloured Progressive Matrices (CPM) | Carlesimo, G. A. (1996). *European Neurology*, *36*, 378-384. |
|  |  |
| Backward Digit Span | Monaco, M. et al. (2013). *Neurological Sciences*, *34*, 749-754. |
| Backward Corsi Span | Monaco, M. et al. (2013). *Neurological Sciences*, *34*, 749-754. |
| Stroop Test (Short Form) | Caffarra, P. et al. (2002). *Nuova Rivista di Neurologia*, *12*, 111-115. |
| **Language** |  |
| Phonemic Verbal Fluency | Costa, A. et al. (2014). *Neurological Sciences*, *35*, 365-372. |
| Semantic Verbal Fluency | Costa, A. et al. (2014). *Neurological Sciences*, *35*, 365-372. |
| **Social cognition** |  |
| Story-Based Empathy Task (SET) | Dodich, A. et al. (2015). *Neurological Sciences*, *36*, 1907-1912. |
| **Attention** |  |
| Digit Cancellation Task | Spinnler, H., & Tognoni, G. (1987). *The Italian Journal of Neurological Sciences*, *6*, S1-S120. |
| Trail-Making Test-A | Giovagnoli, A. et al. (1996). *The Italian Journal of Neurological Sciences*, *17*, 305-309. |
| Trail-Making Test-B | Giovagnoli, A. et al. (1996). *The Italian Journal of Neurological Sciences*, *17*, 305-309. |
| Trail-Making Test-B-A | Giovagnoli, A. et al. (1996). *The Italian Journal of Neurological Sciences*, *17*, 305-309. |
| **Memory** |  |
| Rey Auditory-Verbal Learning Test (RAVLT) | Carlesimo, G. A. (1996). *European Neurology*, *36*, 378-384. |
| Rey-Osterrieth Complex Figure (ROCF) - Delayed Recall | Caffarra, P. et al. (2002). *Neurological Sciences*, *22*, 443-447. |
| Forward Digit Span | Monaco, M. et al. (2013). *Neurological Sciences*, *34*, 749-754. |
| Forward Corsi Span | Monaco, M. et al. (2013). *Neurological Sciences*, *34*, 749-754. |
| **Visuo-spatial abilities** |  |
| Design Copy | Spinnler, H., & Tognoni, G. (1987). *The Italian Journal of Neurological Sciences*, *6*, S1-S120. |
| Rey-Osterrieth Complex Figure (ROCF) – Immediate Copy | Caffarra, P. et al. (2002). *Neurological Sciences*, *22*, 443-447. |
| **Behaviour** |  |
| Edinburgh Cognitive and Behavioural ALS Screen-Behaviour Screen (ECAS) | Poletti, B. et al. (2016). *Amyotrophic Lateral Sclerosis and Frontotemporal Degeneration*, *17*, 489-498. |
| Hospital Anxiety and Depression Scale (HADS) | Costantini, M. et al. (1999). *Supportive Care in Cancer*, *7*, 121-127. |
